# What is a clinical pathway? Refinement of an operational definition to identify clinical pathway studies for a Cochrane systematic review

**DOI:** 10.1186/s12916-016-0580-z

**Published:** 2016-02-23

**Authors:** Adegboyega K. Lawal, Thomas Rotter, Leigh Kinsman, Andreas Machotta, Ulrich Ronellenfitsch, Shannon D. Scott, Donna Goodridge, Christopher Plishka, Gary Groot

**Affiliations:** College of Pharmacy and Nutrition, University of Saskatchewan, Saskatoon, Canada; University of Tasmania and Tasmanian Health Organisation (North), Launceston, Tasmania Australia; Department of Anesthesiology, Sophia Children’s Hospital, Erasmus Medical Centre Rotterdam, Rotterdam, Netherlands; University Medical Center Mannheim, Medical Faculty Mannheim of the University of Heidelberg, Department of Surgery, Mannheim, Germany; Faculty of Nursing, University of Alberta, Edmonton, Alberta Canada; College of Medicine, University of Saskatchewan, Saskatoon, Canada

**Keywords:** Clinical pathways, Quality health improvement, Systematic reviews

## Abstract

Clinical pathways (CPWs) are a common component in the quest to improve the quality of health. CPWs are used to reduce variation, improve quality of care, and maximize the outcomes for specific groups of patients. An ongoing challenge is the operationalization of a definition of CPW in healthcare. This may be attributable to both the differences in definition and a lack of conceptualization in the field of clinical pathways. This correspondence article describes a process of refinement of an operational definition for CPW research and proposes an operational definition for the future syntheses of CPWs literature. Following the approach proposed by Kinsman et al. (BMC Medicine 8(1):31, 2010) and Wieland et al. (Alternative Therapies in Health and Medicine 17(2):50, 2011), we used a four-stage process to generate a five criteria checklist for the definition of CPWs. We refined the operational definition, through consensus, merging two of the checklist’s criteria, leading to a more inclusive criterion for accommodating CPW studies conducted in various healthcare settings. The following four criteria for CPW operational definition, derived from the refinement process described above, are (1) the intervention was a structured multidisciplinary plan of care; (2) the intervention was used to translate guidelines or evidence into local structures; (3) the intervention detailed the steps in a course of treatment or care in a plan, pathway, algorithm, guideline, protocol or other ‘inventory of actions’ (i.e. the intervention had time-frames or criteria-based progression); and (4) the intervention aimed to standardize care for a specific population. An intervention meeting all four criteria was considered to be a CPW. The development of operational definitions for complex interventions is a useful approach to appraise and synthesize evidence for policy development and quality improvement.

## Background

The optimization of patient safety and quality in healthcare remains the primary focus of quality improvement initiatives [1]. The health quality improvement movement has stimulated researchers and managers in healthcare to be innovative in developing new ideas to address issues relating to patient safety and sub-standard care [2, 3]. Several quality improvement concepts, such as Lean, Plan-Do-Study-Act cycles, Continuous quality improvement, etc., and tools such as clinical pathways (CPWs), surgical checklists, etc., have been implemented in a variety of healthcare settings to sustain and support these continuous quality improvement concepts.

CPWs are a common component of these improvement initiatives. They aim to organize and standardize care processes, thus maximizing patient outcomes and improving organization efficiency [4, 5]. Originating in the USA, CPWs have been used in healthcare since the 1980’s. Their goal is to improve patient outcomes, such as mortality rate and others, while containing costs and without compromising quality [6, 7]. Different terms, including care maps, critical pathways, local protocols or algorithms are used to describe the construct of CPWs and may be developed as paper-based or electronic documents [8]. The widespread use and prevalence of CPWs in hospitals is reported in the USA, Australia, Canada, Europe, and Asia [5, 9]. In Canada, CPWs are viewed as a patient-informed knowledge translation tool to ensure clients receive the best available care [10, 11]. The myriad of terms used to describe a CPW has led to conceptual confusion in the field of pathway research.

### Aim and purpose of this paper

The present paper aims to (1) describe the process of refinement and rigorous testing used to obtain an ‘operational definition’ for CPW research, and to (2) propose an operational definition for future syntheses of CPWs literature.

This paper builds on the initial evidence, appraised by Kinsman et al. [7], where a team of Cochrane review authors developed a set of criteria for the practical operational definition of a CPW.

### Purpose and conceptualization of CPWs

CPWs are mainly implemented for specific groups of patients meeting a pre-specified criterion. The implementation of CPWs may be driven by a variation in the quality of care and outcomes for patients with similar health conditions: cardiovascular, respiratory, surgical, cancer, etc. Usually, the aim of the implementation is to reduce a pre-identified variation in patient outcomes and costs and, more recently, to keep patients and families informed about their course of treatment or care.

There are several challenges to CPW research, similar to other complex health service implementation efforts such as conceptualization, implementation, evaluation, and sustainability [8, 12]. De Blesser et al. [13] identified 37 primary definitions for CPWs used in the literature and various terms have been used to describe CPWs in different health settings. There is not a standard definition to identify CPW studies. This paper aims to fill that gap, by proposing a method for the development and refinement of an operational definition for CPW research in healthcare. Clarity of the CPW concept, especially for research, is pivotal to the development of an evidence base; a base that policymakers, healthcare professionals, patients, and other front-line users can refer to for rational decision-making.

### Working definition on CPWs

In 2010, a team of Cochrane authors developed an operational definition for CPWs. The definition can be used to appraise and synthesize international literature on CPWs in hospitals [7]. From three seminal articles [13–15] on CPWs, five operational criteria for CPW definition were rigorously developed [5, 7]. The criteria were subsequently tested on 10 CPWs articles among the review team in a two-stage process. After achieving 100 % agreement, they were applied in the identification of relevant articles for a Cochrane systematic review on CPWs in hospitals published in 2010 [5]. Recent attempts to apply the criteria to CPWs studies conducted in primary care was problematic due to poor reporting of the CPW intervention in the literature. A modification of the original five operational criteria was proposed and agreed by all review authors. This change increased the sensitivity of the operational definition to accommodate relevant literature on CPWs, spanning across a broader context of healthcare settings, including primary care.

## Methods

### Developmental process and refinement of an operational definition for CPWs

In 2010, a team of Cochrane review authors undertook a four-stage process to develop a list of criteria to generate an operational definition for CPWs in hospitals. This followed a methodology proposed by Kinsman et al. [7] and Wieland et al. [16]. The process required (1) identification of articles exploring the scope and definition of CPWs (or similar terms); (2) synthesis of previously suggested components and generation of draft criteria for testing; (3) pilot testing the level of agreement between review authors when applying criteria to identified studies; and (4) modification of the criteria to maximize agreement between review authors [7].

The rigorous testing of the criteria with 10 CPW articles, and with 100 % agreement among review authors, led to the development of a practical operational definition for CPWs: (1) the intervention was a structured multidisciplinary plan of care; (2) the intervention was used to channel the translation of guidelines or evidence into local structures; (3) the intervention detailed the steps in a course of treatment or care in a plan, pathway, algorithm, guideline, protocol or other ‘inventory of actions’; (4) the intervention had timeframes or criteria-based progression (that is, steps were taken if designated criteria were met); and (5) the intervention aimed to standardize care for a specific clinical problem, procedure or episode of healthcare in a specific population [7].

An intervention was considered to be a CPW if it met the first criterion together with three of the other four criteria. The operational definition further supported the identification of relevant full text studies that were eventually used in finalizing the first systematic review on CPWs in hospital care in 2010 [7].

### Rationale for refinement of the operational definition for CPWs

A recent attempt by the review team to conduct a systematic review on CPWs in primary care was hampered by the challenge of applying the above operational definition in a primary care setting, with the major problem during the protocol development for the systematic review [17] being the identification of relevant CPW studies in a primary care context.

This is due to the requirements of criteria numbers 3 and 4. The application of these two criteria is problematic, because several articles explicitly met criterion 3 but not criterion 4, or vice versa, and thus had to be excluded.

TR and LK pilot tested the new criteria on five CPWs studies [18–22] in primary care identified during the protocol development for a systematic review on CPWs in primary care [17]. Consensus was reached among review authors that, by merging the two criteria, the definition would be more inclusive. Thus, the new operational definition for CPWs was narrowed to a four criteria checklist.

## Results

### Pilot test of the new operational definition for consistency

Two review authors (CP and AB) independently pilot-tested the refined operational definition containing the four criteria checklist, with LA serving as an arbitrator to resolve any disagreement during the process. The two review authors had no contact during the pilot test. The pilot test was conducted on 20 articles selected randomly from the 27 included articles from the 2010 Cochrane systematic review on CPWs in hospitals. The aim of the pilot-test was to ensure that the number of articles retrieved for the full text extraction phase in 2010 remained unchanged when using the modified criteria. The results of the pilot-test were collated and a reliability analysis for qualitative variables was estimated using the kappa statistic [23]. Statistical analysis was conducted using SPSS V. 22 (SPSS, Chicago, IL). Table 1 depicts the data layout for the statistical analysis.

**Table 1:** Observed and expected percentage agreement; data layout

	CP		
AB	Include	Pending	Totals
Include	17^a^	0^b^	17^m1^
Exclude	0^c^	3^d^	3^m0^
Totals	17^n1^	3^n0^	20^n^

(a) and (d) represent the number of times the two observers agree while (b) and (c) represent the number of times the two observers disagree, m_1_ = row total number for inclusions, m_0_ = row total number of exclusions,n_1_ = column total for inclusions, n_0_ = column total for exclusions

$$ \mathrm{Observed}\ \mathrm{agreement} = 17/20 = 85\% $$

$$ \mathrm{Expected}\ \mathrm{agreement}\ \left(\mathrm{p}\mathrm{e}\right) = \left[\left(\mathrm{n}1\ /\mathrm{n}\right)\ *\ \left(\mathrm{m}1\ /\mathrm{n}\right)\right] + \left[\left(\mathrm{n}\mathrm{o}\ /\mathrm{n}\right)\ *\ \left(\mathrm{m}\mathrm{o}\ /\mathrm{n}\right)\right] $$

$$ \mathrm{P}\mathrm{e} = \left[\left(17/20\right)\ *\ \left(17/20\right)\right] + \left[\left(3/20\right)\ *\ \left(3/20\right)\right] = 0.722 + 0.023 = 0.75 $$

The first independent pilot testing, applying the new criteria to the previously published CPW articles described above, generated 85 % observed and 75 % expected agreement, respectively (Table 2). For the reliability analysis, the kappa test statistic was 0.99 with a *P* value <0.001, implying perfect agreement between the two reviewers (Table 3).

**Table 2:** Inter-rater reliability analysis of 20 articles on clinical pathways in hospital care; data layout

AB * CP		Cross-tabulation		
Count				
		CP		Total
		1.00	0.00	
AB	1.00	17	0	17
	0.00	0	3	3
Total		17	3	20

AB, reviewer 1; CP, reviewer 2; 1.00, Included; 0.00, Pending

**Table 3:** SPSS output for Kappa statistic

Symmetric measures					
		Value	Asymp. Std. Error^a^	Approx. T^b^	Approx. Sig.
Measure of Agreement	Kappa	1.000	0.000	4.472	0.000
N of Valid Cases		20			

^a^Not assuming the null hypothesis

^b^Using the asymptotic standard error assuming the null hypothesis

Approx. Sig. = *P* value

### Refined criteria for CPW operational definition

The four criteria derived from the refinement process described above are (1) Is it a structured multidisciplinary care plan? (2) Is it used to channel the translation of guidelines or evidence into local structures? (3) Does it detail the steps in a course of treatment or care in a plan, pathway, algorithm, guideline, protocol or other ‘inventory of actions’ (i.e. the intervention had time frames or criteria based progression)? (4) Does it aim to standardize care for a specific clinical problem, procedure or episode of care in a specific population?

Henceforth, an intervention was considered a CPW if it contained all of the four criteria in the new operational definition. Subsequently, the new definition will be applied to identify (1) relevant titles and abstracts for an on-going update of a Cochrane systematic review on CPWs in hospitals, and (2) a new Cochrane systematic review on CPWs in primary care.

Prospectively, the systematic review will follow the validated Cochrane Effective Practice and Organization of Care (EPOC) methodology for complex interventions, and will consider randomized controlled trials, non-randomized controlled trials, controlled before and after, and interrupted time series study designs [18].

## Discussion

This article describes the process of development, refinement, and testing of a practical working definition for CPWs. It also supports the inclusion of relevant primary research articles only in our Cochrane systematic review update on CPW effectiveness in hospital and primary care. The initial criteria, rigorously developed in 2010 using the Kinsman et al. [7] and Wieland et al. [16] approach for a Cochrane systematic review, is a milestone in the field of CPW research. The first proposed operational definition of a CPW ultimately led to the successful completion of the first Cochrane EPOC review on CPWs in hospitals.

It is imperative to understand that we created an operational definition for CPWs in hospital and primary care settings, rather than undergoing a scientific concept analysis [24]. We propose the development of minimum inclusion criteria and an operational definition for systematic reviews of complex interventions such as CPWs. This is due to the high level of resources required of Walker and Avant’s gold standard concept analysis process and the likelihood that the required expertise and time required may not be feasible for healthcare decision makers or implementers. Our belief is that this approach serves as a preliminary step to ensure all-important studies are catalogued while simultaneously including only the relevant evidence. Future work will be conducted to ascertain the sensitivity of the refined criteria in identifying pathway studies in primary care.

Although it has been established in previous literature that CPWs are complex interventions, this information is not sufficient and useful for the development of an evidence base for CPWs in the international literature. There is variation in the terms used to depict a CPW, therefore referring to a CPW as a complex intervention without standardizing its elements only adds to complexity and confusion towards the attainment of a standard definition. The pilot test for the operational criteria and reliability analysis shows a significant high level of agreement among reviewers. This demonstrates that the resulting criteria have the potential to be clear and objective enough to permit further research relevant to the field of CPWs. This methodology may be refined and applied to similar fields also challenged with the issue of cataloguing and reviewing the evidence for complex health service interventions.

An internationally agreed operational definition for CPWs will (1) limit the confusion regarding CPWs, interventions that have been attributed to a naming system in different contexts; (2) ultimately generate a positive discourse among researchers and frontline providers; (3) provide an adequate understanding of what actually constitutes a CPW; and (4) will permit the utilization of rigorous designs, such as realist and systematic reviews, to appraise effectiveness, contextual factors, and mechanisms that may further improve CPW implementation in different healthcare settings.

Employing appropriate implementation theories and taking into account the level of implementation will enhance the long-term sustainability and uptake of CPWs in healthcare. This will lead to a better understanding of how CPWs may contribute to better healthcare.

## Conclusions

Worldwide CPW implementation and usage in healthcare is on the rise. The lack of an agreed-upon definition of a CPW and what is not a CPW remains a significant challenge for many CPW researchers and clinicians. This paper describes the process of developing, refining, and pilot testing a set of criteria to be used for a practical operational definition for CPWs in healthcare, following the Kinsman et al. [7] and Wieland et al. [16] approach.

Future researchers considering the development, implementation, and evaluation of CPWs should adopt the developed definition or modify them to fit into their future research. This will advance the discourse towards an internationally agreed-upon definition of what constitutes a CPW in healthcare.
